# Influence of trigger type, tube voltage and heart rate on calcified plaque imaging in dual source cardiac computed tomography: phantom study

**DOI:** 10.1186/1471-2342-14-30

**Published:** 2014-09-01

**Authors:** Tobias Penzkofer, Eva Donandt, Peter Isfort, Thomas Allmendinger, Christiane K Kuhl, Andreas H Mahnken, Philipp Bruners

**Affiliations:** 1 Department of Diagnostic and Interventional Radiology, Aachen University Hospital, RWTH Aachen University, Pauwelsstr. 30, 52074 Aachen, Germany; 2 Surgical Planning Laboratory, Department of Radiology, Brigham and Women’s Hospital, 75 Francis Street, 02115 Boston, USA; 3 Applied Medical Engineering, Helmholtz-Institute Aachen, RWTH Aachen University, Pauwelsstr. 20, 52074 Aachen, Germany; 4 Siemens Healthcare, CT Division, Forchheim, Germany; 5 Department of Diagnostic and Interventional Radiology, University Hospital Marburg, Philipps University of Marburg, Marburg, Germany

## Abstract

**Background:**

To investigate the impact of high pitch cardiac CT vs. retrospective ECG gated CT on the quantification of calcified vessel stenoses, with assessment of the influence of tube voltage, reconstruction kernel and heart rate.

**Methods:**

A 4D cardiac movement phantom equipped with three different plaque phantoms (12.5%, 25% and 50% stenosis at different calcification levels), was scanned with a 128-row dual source CT scanner, applying different trigger types (gated vs. prospectively triggered high pitch), tube voltages (100-120 kV) and heart rates (50–90 beats per minute, bpm). Images were reconstructed using different standard (B26f, B46f, B70f) and iterative (I26f, I70f) convolution kernels. Absolute and relative plaque sizes were measured and statistically compared. Radiation dose associated with the different methods (gated vs. high pitch, 100 kV vs. 120 kV) were compared.

**Results:**

Compared to the known diameters of the phantom plaques and vessels both CT-examination techniques overestimated the degrees of stenoses. Using the high pitch CT-protocol plaques appeared larger (0.09 ± 0.31 mm, 2 ± 8 percent points, PP) in comparison to the ECG-gated CT-scans. Reducing tube voltage had a similar effect, resulting in higher grading of the same stenoses by 3 ± 8 PP. In turn, sharper convolution kernels lead to a lower grading of stenoses (differences of up to 5%). Pairwise comparison of B26f and I26f, B46f and B70f, and B70f and I70f showed differences of 0–1 ± 6–8 PP of the plaque depiction. Motion artifacts were present only at 90 bpm high pitch experiments. High-pitch protocols were associated with significantly lower radiation doses compared with the ECG-gated protocols (258.0 mGy vs. 2829.8 mGy CTDI_vol_, p ≤ 0.0001).

**Conclusion:**

Prospectively triggered high-pitch cardiac CT led to an overestimation of plaque diameter and degree of stenoses in a coronary phantom. This overestimation is only slight and probably negligible in a clinical situation. Even at higher heart rates high pitch CT-scanning allowed reliable measurements of plaque and vessel diameters with only slight differences compared ECG-gated protocols, although motion artifacts were present at 90 bpm using the high pitch protocols.

## Background

Cardiac computed tomography (CT) is an established non-invasive method of assessing coronary artery morphology both in emergency and routine settings [1]. It is superior to magnetic resonance based methods of coronary angiography with respect to temporal and spatial resolution. However, continuous motion of the heart makes cardiac cross-sectional imaging a challenging task [1,2]. Different methods have been developed to overcome this problem, many of them rely on the uniformity of the cardiac cycle by imaging different sections of the heart over several consecutive heart beats. To date the vast majority of current CT systems uses either a prospectively ECG-triggered or retrospectively ECG-gated scanning protocol for this purpose. Since all these methods are associated with ionizing radiation applied to the patient, the risks and benefits need to be carefully weighted [3].

Recent advances in CT technology led to the development of dual source based high pitch (pitch up to 3.4) scanning protocols with table speeds of up to 46 cm/sec [4-6], or even higher [7,8]. These protocols promise to image the heart within one heartbeat, obviating the need for gating or multi-step triggering, i.e. acquisition techniques that are inherently associated with redundant scan ranges leading to increased radiation doses.

The goal of this study was to evaluate the quantification of calcified stenosis in high pitch cardiac CT-scans versus an ECG-gated scan protocol in a controlled phantom environment. Secondary goal was to evaluate the influence of tube current, convolution kernel and heart rate on the different acquisition protocols.

## Methods

### Coronary movement phantom

**Figure 1:**
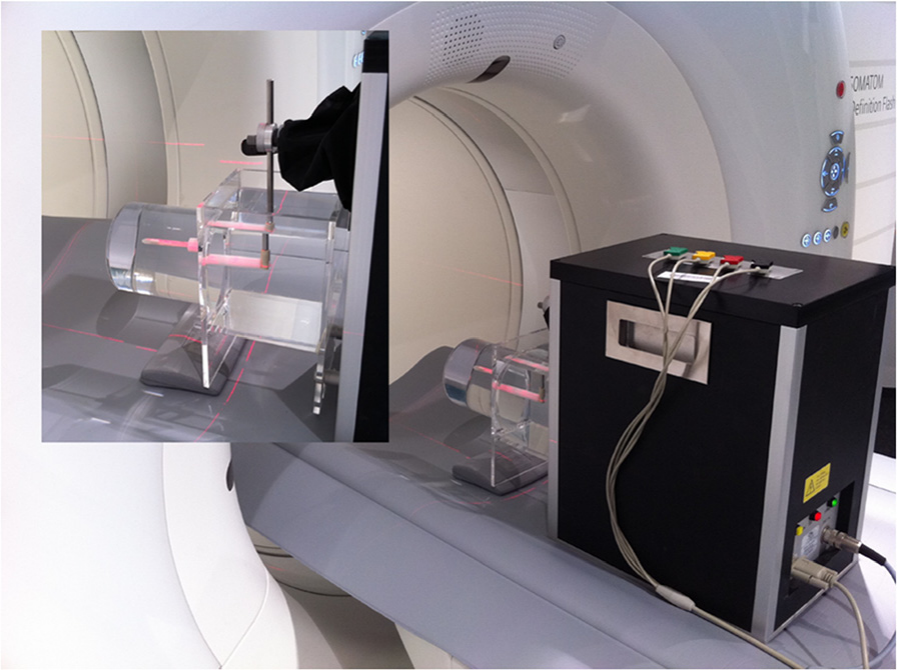
**Cardiac movement phantom setting during the scans.** (inlay) the coronary artery phantom (CAP) was placed in a water filled container. The cardiac phantom provides 4D motion with ECG-syncing over the scanners standard ECG-interface (ECGI).

**Figure 2:**
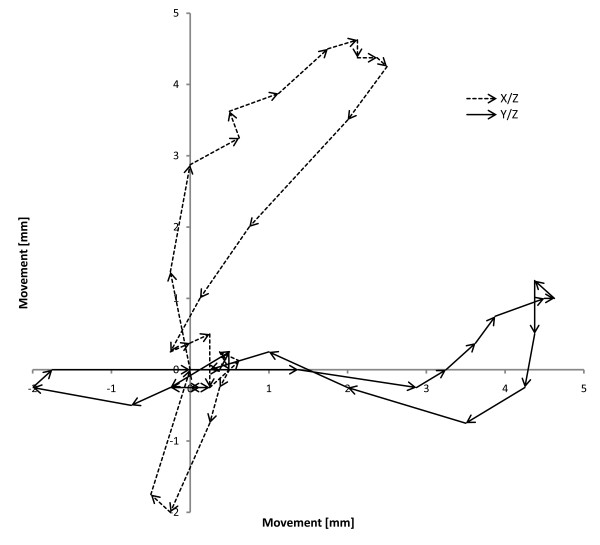
**Cardiac movement as performed by the coronary movement simulator.** The solid line represents the movement in the Y/Z plane, while the dashed line shows the path in the X/Z plane. Arrow length corresponds to movement speed.

A 4D coronary movement simulator (Sim4Dcardiac, QRM GmbH, Möhrendorf, Germany, Figure 1) was used for the study performing coronary artery movement patterns with heart rates of 50, 70 and 90 beats per minute (bpm). Three custom made dedicated coronary artery phantoms (QRM GmbH, Möhrendorf, Germany) each with defined calcified (high, medium and low calcification at 796, 401 and 197 mgHA/cm^3^ at densities of 1.58, 1.30 and 1.16 g/cm^3^) coronary stenoses of various degrees (50%, 25% and 12.5%) were used in a simulated 4.0 mm vessel. The absolute stenosis sizes were 0.5 mm (12.5%), 1 mm (25%) and 2 mm (50%). The movement protocol was derived from an electron beam scan (Figure 2) [9]. The coronary simulator provides an ECG output mapping the movement pattern to an ECG signal readable by the scanner’s ECG analysis module.

### CT examination protocols

A 128 slice high pitch capable dual source computed tomography scanner (Siemens Somatom Flash, Siemens Healthcare, Forchheim, Germany) was used for all experiments. The high pitch scans were performed using a dedicated high pitch cardiac protocol (dual source, 100 and 120 kV, 320 mAs/rot, pitch 3.4, prospective ECG trigger, collimation 128 × 0.6 mm, FoV 190 × 190 mm, scan length 90.0 mm, rotation time 0.28s) and a retrospectively ECG gated protocol (100 and 120 kV, 320 mAs/rot, retrospective ECG gating after pulsing at 50-100%, collimation 128 × 0.6 mm, pitch 0.19, rotation time 0.28s). Reconstructions were performed according to the vendor’s specifications, identically for both modes with B26f, B46f, B70f standard kernels and iterative I26f and I70f kernels with a field of view of 190 × 190 mm and slice thicknesses of 0.6 mm (B26f, B46f, B70f) and 0.75 mm (I26f, I70f) avoiding undersampling of the acquired data.

### Measurements

Measurements were performed manually using dedicated DICOM viewer software (Synedra View, Version 3.1, Synedra GmbH, Aachen, Germany) by one observer, and checked for validity by two others with 3 and 5 years of experience in thoracic imaging. For each combination of Kernel/Method/kV/plaque phantom three repeated measurements of plaque diameter and three repeated measurements of total vessel diameter were performed resulting in a total of 2,880 data points. The repeated measurements were averaged and used for statistical analyses.

### Statistical analyses

Statistical analysis was performed using SPSS 19 (SPSS Inc., Chicago, Illinois, USA) and MedCalc (MedCalc Software, Mariakerke, Belgium). Tests performed were Analysis of Variances with post-hoc testing and Bland-Altman as well as Mountain plot method comparisons. P-values of 0.05 or lower were considered statistically significant, multiple testing correction (Bonferroni) was performed where applicable. Additional tests were performed using non-parametric Mann–Whitney-U analyses.

## Results

**Figure 3:**
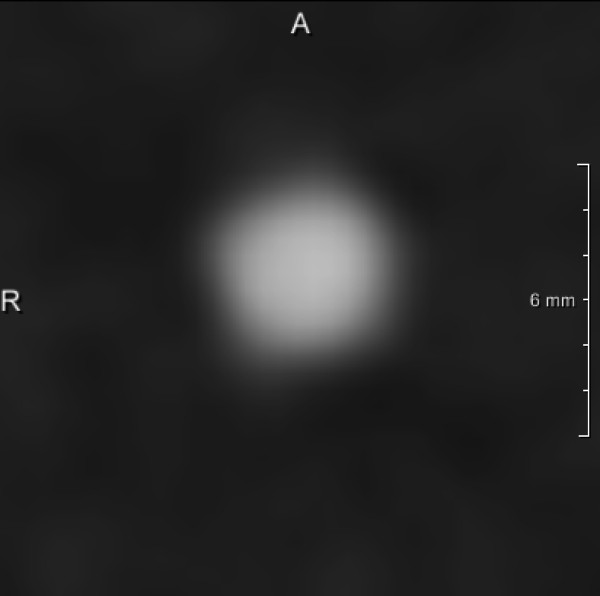
**Low calcified stenosis (197 mmHA/cm**^**3**^**) at the same position as Figure**8**; the stenosis grade is not distinguishable due to insufficient contrast.**

The plaque phantom featuring the lowest calcification level (197 mmHA/cm^3^) was not measureable with the applied methods due to low contrast between plaque and vessel lumen (Figure 3). The following data result from the measurements of the intermediately (401 mmHA/cm^3^) and heavily (796 mmHA/cm^3^) calcified plaque phantoms.

### Comparison to phantom dimensions

In comparison to the phantom vessel dimensions retrospectively ECG-gated and prospectively ECG-triggered high pitch scanning provided vessel diameters of between 3.93 ± 0.2 mm and 4.70 ± 0.5 mm with a tendency to a slight overestimation (Table 1). The plaque thickness was measured at between 0.88 ± 0.1 mm and 1.27 ± 0.1 mm (0.5 mm/12.5%), 1.40 ± 0.2 mm and 1.80 ± 0.4 mm (1 mm/25%) and 1.98 ± 0.2 mm and 2.39 ± 0.4 mm (2 mm/50%) again showing an overestimation for both cardiac CT techniques which was more pronounced for high-pitch scanning. These findings led to the following degrees of stenoses: 22.4 ± 3.3% and 29.2 ± 2.4% (12.5% stenoses), 34.9 ± 4.6% and 39.7 ± 5.9% (25% stenoses) and 48.6 ± 4.3% and 59.6 ± 7.1% (50% stenoses) for retrospectively gated and prospectively triggered high pitch, respectively.

**Table 1:** Absolute calcified stenosis diameters (true diameters: 12.5%: 0.5 mm, 25%: 1.0 mm, 50%: 2.0 mm), measured total vessel diameters (true diameter 4.0 mm) as measured for the different protocol types per phantom type

**Density [mmHA/cm**^**3**^**]**	**True stenosis size**	**Method**	**Plaque diameter [mm]**	**Vessel diameter [mm]**	**Measured stenosis [%]**
197	12,5 %	High pitch	-	-	-
	(0.5 mm/4.0 mm)	Gating	-	-	-
	25%	High pitch	-	-	-
	(1.0 mm/4.0 mm)	Gating	-	-	-
	50%	High pitch	-	-	-
	(2.0 mm/4.0 mm)	Gating	-	-	-
401	12,5%	High pitch	0.97 ± 0.1	3.96 ± 0.1	24.6 ± 2.7
	(0.5 mm/4.0 mm)	Gating	0.88 ± 0.1	3.95 ± 0.1	22.4 ± 3.3
	25%	High pitch	1.48 ± 0.2	4.13 ± 0.2	35.8 ± 4.3
	(1.0 mm/4.0 mm)	Gating	1.40 ± 0.2	4.00 ± 0.1	34.9 ± 4.6
	50%	High pitch	1.98 ± 0.2	4.08 ± 0.2	48.6 ± 4.3
	(2.0 mm/4.0 mm)	Gating	1.98 ± 0.2	4.00 ± 0.1	49.6 ± 4.6
796	12,5%	High pitch	1.27 ± 0.1	4.35 ± 0.4	29.2 ± 2.4
	(0.5 mm/4.0 mm)	Gating	1.18 ± 0.1	4.52 ± 0.4	26.2 ± 2.6
	25%	High pitch	1.80 ± 0.4	4.52 ± 0.4	39.7 ± 5.9
	(1.0 mm/4.0 mm)	Gating	1.71 ± 0.4	4.70 ± 0.5	36.1 ± 5.0
	50%	High pitch	2.39 ± 0.4	3.99 ± 0.3	59.6 ± 7.1
	(2.0 mm/4.0 mm)	Gating	2.22 ± 0.3	3.93 ± 0.2	56.3 ± 5.9

The lowest density plaque phantoms (197 mmHA/cm^3^) were not measurable as no sufficient contrast could be established between the lumen and the plaque mimic.

Separated by heart rate of the phantom, both examination protocols showed an overestimation of vessel diameter, plaque diameter and degree of stenosis which was more pronounced for the 12.5% in comparison to the 50% stenosis and for the high pitch protocol in comparison to ECG-gated scanning. Diameter measurements for the same degree of stenosis at different heart rates did not show relevant differences (Table 2).

**Table 2:** Absolute plaque/stenosis diameters (true diameters: 12.5%: 0.5 mm, 25%: 1.0 mm, 50%: 2.0 mm), measured vessel diameters (true diameter 4.0 mm) per heart rate and cardiac CT method

**True stenosis [%]**	**Heart rate [bpm]**	**Method**	**Plaque diameter [mm]**	**Vessel diameter [mm]**	**Stenosis [%]**	**Deviation [PP]**
12.5%	50	High pitch	1.13 ± 0.19	4.17 ± 0.35	27.0 ± 3.5	14.5
		Gating	1.03 ± 0.19	4.23 ± 0.45	24.4 ± 3.7	11.9
	70	High pitch	1.12 ± 0.18	4.14 ± 0.35	27.1 ± 3.4	14.6
		Gating	1.03 ± 0.19	4.26 ± 0.41	24.1 ± 3.6	11.6
	90	High pitch	1.11 ± 0.19	4.15 ± 0.30	26.7 ± 3.5	14.2
		Gating	1.03 ± 0.19	4.22 ± 0.40	24.4 ± 3.4	11.9
25%	50	High pitch	1.63 ± 0.34	4.35 ± 0.38	37.2 ± 5.7	12.2
		Gating	1.53 ± 0.33	4.35 ± 0.49	35.0 ± 4.6	10.0
	70	High pitch	1.65 ± 0.33	4.33 ± 0.36	37.8 ± 5.1	12.8
		Gating	1.53 ± 0.34	4.35 ± 0.51	34.8 ± 4.6	9.8
	90	High pitch	1.66 ± 0.35	4.29 ± 0.39	38.4 ± 5.8	13.4
		Gating	1.61 ± 0.36	4.36 ± 0.51	36.7 ± 5.2	11.7
50%	50	High pitch	2.19 ± 0.37	4.06 ± 0.20	53.9 ± 8.0	3.9
		Gating	2.08 ± 0.28	3.95 ± 0.18	52.7 ± 6.2	2.7
	70	High pitch	2.16 ± 0.36	4.00 ± 0.23	54.0 ± 7.9	4.0
		Gating	2.11 ± 0.27	3.99 ± 0.14	52.9 ± 6.1	2.9
	90	High pitch	2.20 ± 0.38	4.05 ± 0.21	54.4 ± 8.4	4.4
		Gating	2.11 ± 0.32	3.96 ± 0.19	53.1 ± 6.7	3.1

Deviations are given in percentage difference to the true diameters as specified by the phantom manufacturer. (PP: percent points).

### Comparison of trigger types

**Figure 4:**
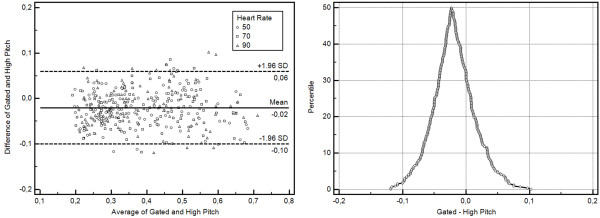
**Method comparisons for the two trigger types under investigation.** Bland-Altman-Plotting reveals a slight overestimation of stenosis percentage (2 ± 8%). Mountain plotting shows no relevant outliers.

High pitch scanning resulted in a larger depiction of the plaques in comparison to the ECG-gated scan method (0.09 ± 0.31 mm). This difference accounted for an additional overestimation of the stenoses by 2 ± 8 percent points (PP) (Table 3). Bland-Altman plotting (Figure 4) revealed this systematic difference, while mountain plotting additionally revealed no outliers and a narrow distribution profile.

**Table 3:** Average difference in degree of stenosis (Δ percentage) and measured plaque diameter (Δ diameter) between the two cardiac CT methods and tube voltages (first vs. second mentioned)

**Comparison**	**Δ Percentage**	**Δ Diameter**
Gated/high pitch	2 ± 8 PP	0.09 ± 0.31 mm
100 kV/120 kV	3 ± 8 PP	0.14 ± 0.32 mm

(PP: percent points).

### Trigger type and heart rate

Comparing the two CT imaging methods with respect to their plaque depiction depending on heart rate, a descending difference of the measured plaque diameter was found with increasing heart rate (50 bpm: 0.1 ± 0.28 mm, 70 bpm: 0.09 ± 0.31 mm and 90 bpm: 0.07 ± 0.35 mm, Table 4). However, the difference was so small at all heart rates that no clinically relevant effect on the degree of stenosis (2 ± 7–8 PP) was observed (Table 4).

**Table 4:** Average difference in degree of stenosis (Δ percentage) and measured plaque diameter (Δ diameter) per heart rate between the two cardiac CT examination protocols (PP: percent points)

**Heart rate**	**Δ Percentage (gating vs. high pitch)**	**Δ Diameter**
50 bpm	2 ± 7 PP	0.10 ± 0.28 mm
70 bpm	2 ± 8 PP	0.09 ± 0.31 mm
90 bpm	2 ± 8 PP	0.07 ± 0.35 mm

### Tube voltage

**Figure 5:**
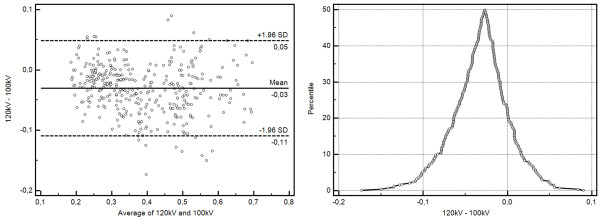
**Method comparison plots for the tube voltage comparison at 100 and 120 kV.** While on average only a slight overestimation for 100 kV is shown, some outliers are present in mountain plotting.

In comparison to 120 kV tube voltage 100 kV CT imaging resulted in a difference of 0.14 ± 0.32 mm regarding plaque diameters. This led to a 3 ± 8 PP overestimation of the stenosis diameter using 100 kV scan protocols (Figure 5, Table 4).

### Reconstruction kernels

**Figure 6:**
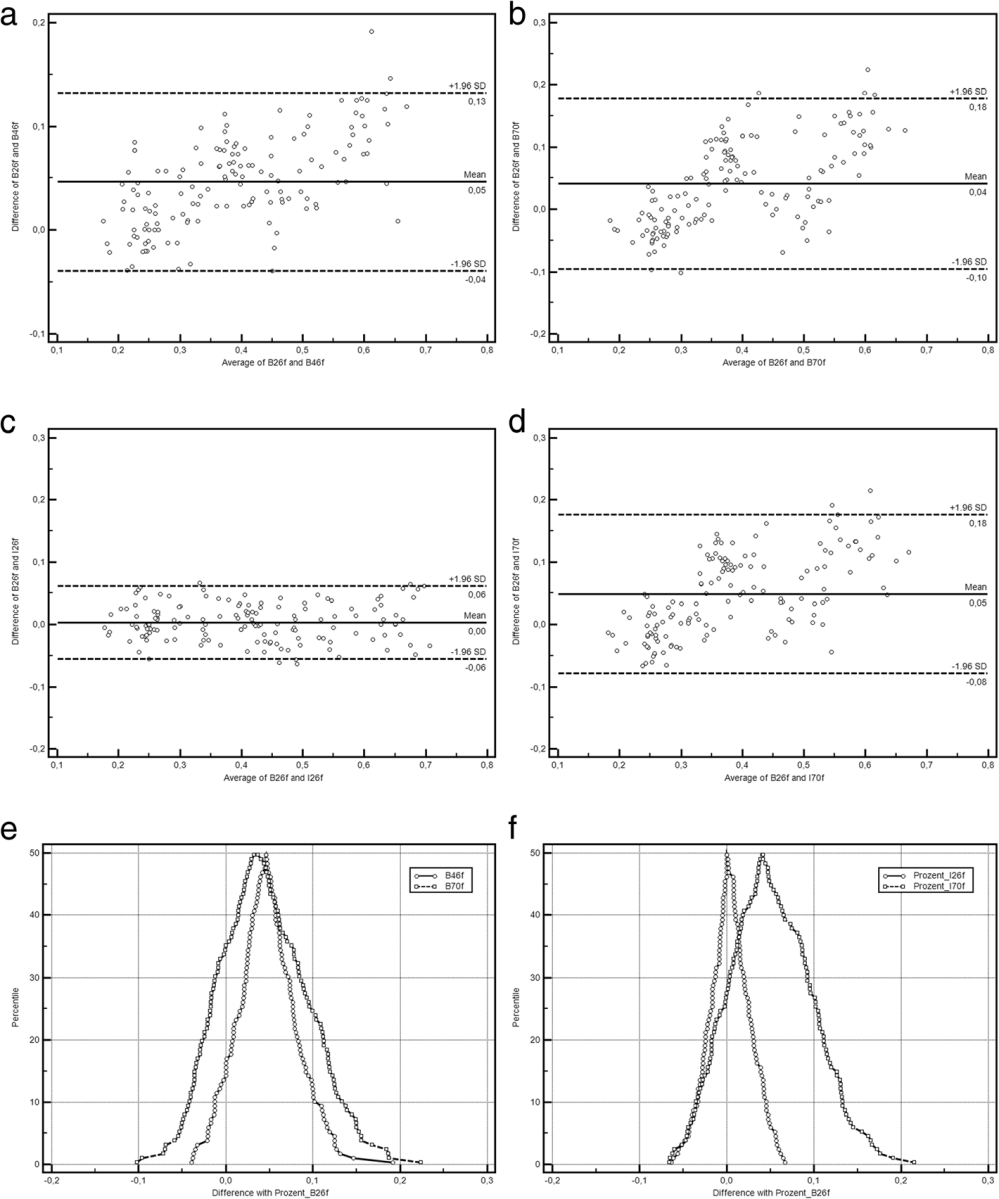
**Method comparison plots for kernel comparisons (a-d Bland-Altman plots, e-f mountain plots).** Shown are the differences between the standard B26f and the other kernels under investigation (**a**, **e**: B46f, **b**, **e**: B70f, **c**, **f**: I26f, **d**, **f**: I70f). While for B26f and I26f align perfectly, skewed distributions with outliers are visible for all other comparisons.

Smooth reconstruction kernels (B26f, I26f) showed a higher deviation from the true plaque diameters in comparison to the sharp kernels (B70f, I70f). This result was even more pronounced for the heavily calcified plaques which were significantly overestimated regarding their diameter when smooth kernels were used for image reconstruction. The B46f kernel exhibited a intermediate performance, with the least deviation in the less calcified plaque and a deviation between the standard and iterative kernels in the heavily calcified setting. The comparison between corresponding standard and iterative reconstruction kernels (B26f vs. I26f; B70f vs. I70f) did not reveal any relevant difference (Table 5, Figure 6).

**Table 5:** Pairwise comparisons of the average difference in degree of stenosis (Δ percentage) and measured plaque diameter for all used kernels

	**796 mmHA/cm**^**3**^		**401 mmHA/cm**^**3**^	
	**Δ Diameter**	**Δ Percentage**	**Δ Diameter**	**Δ Percentage**
B26f	0.88 ± 0.26 mm	15.9 ± 4.2%	0.39 ± 0.27 mm	8.6 ± 6.8%
I26f	0.86 ± 0.25 mm	10.8 ± 5.0%	0.38 ± 0.26 mm	4.3 ± 6.5%
B46f	0.53 ± 0.27 mm	9.2 ± 6.6%	0.18 ± 0.26 mm	6.9 ± 6.9%
B70f	0.36 ± 0.33 mm	15.3 ± 4.0%	0.26 ± 0.27 mm	8.5 ± 6.6%
I70f	0.34 ± 0.33 mm	8.9 ± 6.7%	0.21 ± 0.26 mm	5.8 ± 6.6%

### Radiation dose comparison

Radiation doses were significantly lower for high pitch CT in comparison to ECG gated scanning (258.0 mGy vs. 2829.8 mGy for CTDIvol, 36.3 mGycm vs. 341.2 mGycm for the dose-length-product, DLP), and lower for 100 kV vs. 120 kV scan protocols (962.6 mGy vs. 2125.2 mGy CTDIvol and 115.1 mGycm vs 262.4 mGycm DLP). All these differences were statistically significant (p ≤ 0.0001 for trigger type and p ≤ 0.0005 for tube voltage, Tables 6, 7).

**Table 6:** Dose comparison between the two used cardiac CT methods

**Trigger**	**Gated**	**High pitch**	**Statistics**
CTDIvol [mGy]	2829.8 ± 1539.4	258.0 ± 66.3	p < 0.0001
DLP [mGycm]	341.2 ± 150.7	36.3 ± 9.6	p < 0.0001

Both DLP and CTDIvol are significantly lower for high pitch cardiac CT protocols in comparison to gating.

**Table 7:** Dose comparison between the two used tube voltages

**kV**	**100**	**120**	**Statistics**
CTDIvol [mGy]	962.6 ± 862.9	2125.2 ± 2082.3	p = 0.0005
DLP [mGycm]	115.1 ± 93.4	262.4 ± 225.2	p = 0.0002

Both DLP and CTDIvol are significantly lower for 100 kV in comparison to 120 kV.

### Motion artifacts

**Figure 7:**
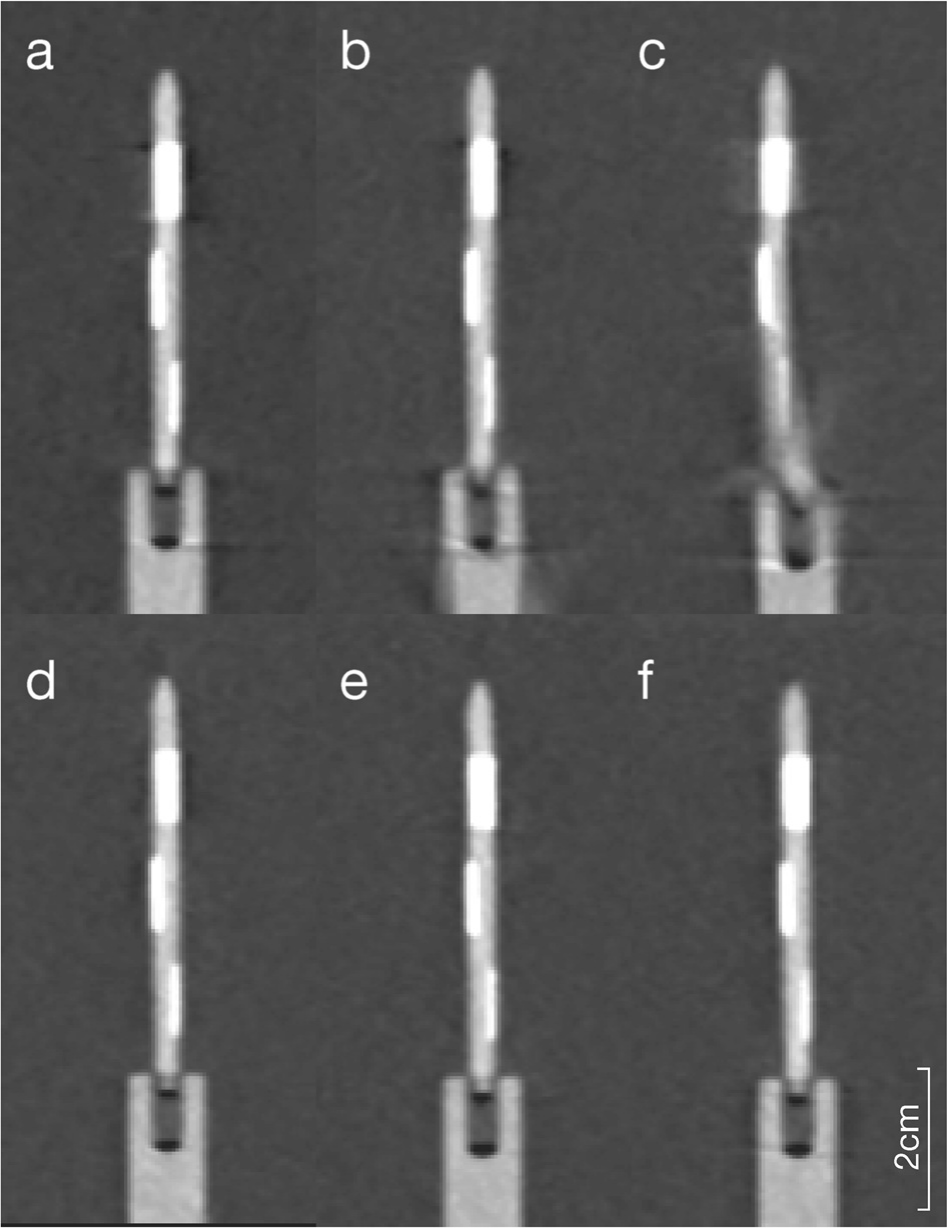
Coronary reformation of the phantom scans (a-c prospectively triggered high-pitch, d-f: retrospective gating) at different heart rate settings (a/d: 50 bpm, b/e: 70 bpm, c/f: 90 bpm).

Coronal reconstruction revealed motion artifacts, present at the proximal end of the coronal phantom for the 90 bpm high pitch prospectively gated scans (Figure 7), which were not present in retrospectively gated scanning or the 50 or 70 bpm high pitch experiments. No other occurrences of motion artifacts were observed.

## Discussion

Despite the fact that coronary CTA has found its way into the clinical workup of patients with suspected coronary artery disease, there are still major concerns regarding the applied radiation dose, which was determined to be in the typically order of 12 mSv (8–18 mSv) [10]. In comparison the effective radiation doses applied during invasive coronary angiography are reported to be approximately 5 mSv [11] with ranges from 2.3 to 22.7 mSv [3]. Because of its ability to rule out hemodynamically relevant coronary stenoses with a high negative predictive value, coronary CTA is especially suited for patients presenting with typical symptoms but having a low pre-test likelihood for coronary artery disease [12]. During the last few years different technical developments have been introduced in clinical routine practice in order to significantly reduce radiation dose of coronary CTA. These techniques include the use of lower tube voltage (e.g. 100 instead of 120 kV) [13] and the application of ECG-dependent tube current modulation. The latter bases upon the reduction of tube current of up to 80% during systolic phase and full dose is only applied during diastolic phase. This approach allows for dose reduction of up to 50% [14]. Another technique resulting in substantial reduction of radiation dose is realized by the use of prospective ECG-triggered transverse data acquisition (also known as “step and shot acquisition”) instead of retrospective gated spiral scanning [15].

With the introduction of a modern dual-source CT scanner with simultaneous acquisition of 64 slices and a gantry rotation time of 330 ms a temporal resolution of 83 ms became technically feasible [16]. Latest generation of dual-source scanners reduced the rotation time to 0.28 s. Furthermore, due to the ca. 95° offset of both x-ray tubes within the gantry of dual source CT in combination with fast and precise table movement the use of high-pitch (>3.0) scan protocols for coronary CTA could be performed. The result was the ability to cover the whole heart within a single cardiac cycle [17]. Since then, different studies showed the diagnostic value of prospectively ECG-triggered high-pitch spiral coronary CTA [18,19].

The aim of the presented study was to investigate influence of scan protocol in combination with ECG-synchronization (prospective triggered high-pitch vs. retrospective ECG-gated spiral CT), tube voltage (100 vs. 120 kV) and heart rate (50, 70, 90 bpm) on quantification of coronary artery stenosis due to plaques with different calcification levels. In general, all used CT examination protocols resulted in an overestimation of stenoses which increased with increasing calcium content and decreased with increasing grade of stenoses. This overestimation was slightly higher for the high pitch examination protocols in comparison to the retrospective ECG-gated technique. Both observations (overestimation of small lesions, which are highly calcified) are mainly due to blooming artifacts which occur when sharp edges with high attenuation differences are encountered in the scan volume [20,21]. A reason for the slight worsening of this phenomenon, when high pitch examination protocols are used, may be the fact that attenuation data of two separate detectors are used for image reconstruction. However, in the clinical situation this finding may be of little relevance due to a maximum difference of 3 percent points regarding grade of stenosis (small, highly calcified plaque).

**Figure 8:**
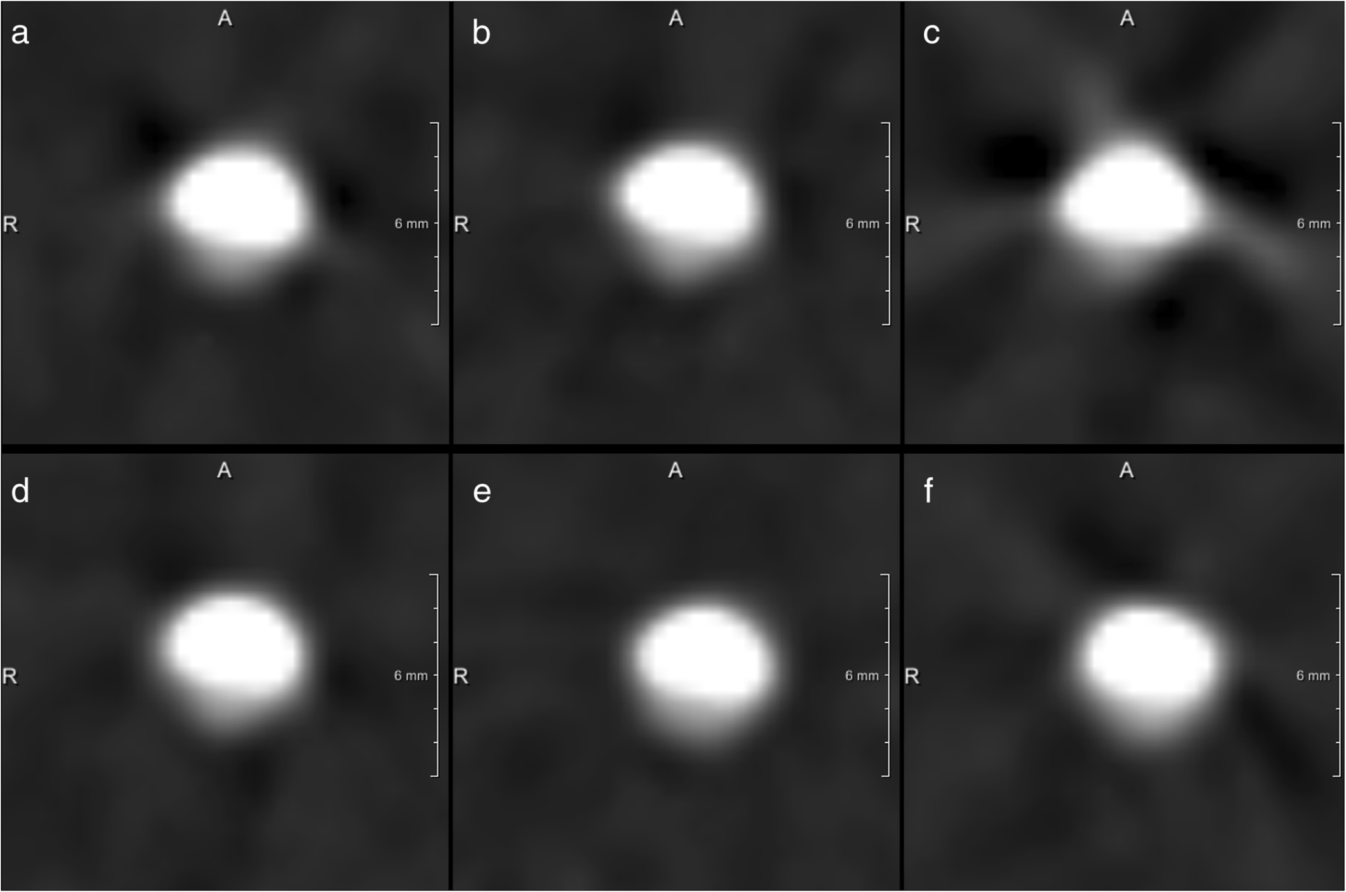
Image data reconstructed in different heart rate settings (a/d: 50 bpm; b/e: 70 bpm; c/f: 90 bpm, B26f) using either prospectively ECG-triggered high-pitch scanning (a – c) or retrospectively ECG-gated cardiac CT (d – f).

In most of the recently published clinical studies on prospective ECG-triggered high pitch cardiac CT a heart rate more than 60 bpm was defined as an exclusion criterion [4,18]. In our experimental setup there were no significant differences regarding vessel diameter, plaque diameter and grade of stenoses between the different heart rates (50, 70, 90) although we found a slight trend to larger deviations of the measured parameters comparing the 50 and the 90 bpm data (Table 2). The motion artifacts observed at 90 bpm high pitch scanning should however raise concerns about the applicability of the technique at such heart rates (Figure 8). This finding is at least to some degree in contrast to clinical findings which were recently published [22]. Scharf et al. reported about a cohort of 111 consecutive patients who underwent prospectively ECG-triggered high-pitch spiral CT of the chest for non-cardiac reason. The evaluation of image quality showed a significant difference of mean heart rate and mean heart rate variability between patients with diagnostic and non-diagnostic images of the coronary arteries. The optimal values were calculated with a heart rates lower than 64 bpm and heart rate variabilities of less than 13 bpm. With respect to this result it needs to be discussed that we used an extremely reliable experimental setup providing an absolutely stable heart rate. Nevertheless, our results may be a hint that prospectively ECG-triggered high-pitch cardiac CT may also be suited for patients presenting stable heart rates > 60 bpm and a low heart rate variability. This hypothesis is supported by the results of Feuchtner et al. who found significantly higher diagnostic image quality in patients with stable sinus rhythm but without premedication for heart rate control in comparison to patients with arrhythmia using a prospective triggered “step and shoot” CT-examination mode [23]. In this study a mean heart rate of 66.2 ± 8 bpm in patient group with stable sinus rhythm was associated with only 0.5% non-diagnostic coronary segments whereas 4% of coronary segments were scored non-diagnostic in the arrhythmia group with a mean heart rate of 70 ± 15 bpm.

In our experimental setting the use of a prospectively ECG-triggered high-pitch spiral examination protocol resulted in a reduction of radiation exposure of approximately 90% in comparison to a retrospective ECG-gated cardiac CT-examination (Table 6). This finding is mainly due to the significant oversampling using a pitch of 0.19 for retrospective ECG-gating in order to acquire enough data in all cardiac phases. On the other hand this allows quantification of cardiac function in addition to the evaluation of coronary arteries. Comparing the measured diameters for the 100 and 120 kV examination modes we found a slight overestimation for the 100 kV CT-protocol (Table 3). However, we consider the differences (3 ± 8%) clinically not relevant whereas reduction of tube voltage results in further reduction of radiation dose applied to the patient. Due to the fact that lower tube voltage leads to increased image noise some authors used 100 kV scan protocols in patients < 100 kg body weight and 120 kV for > 100 kg, respectively [24].

Due to the experimental design of the study our results cannot be directly transferred into the clinical situation. The employed phantom provides a stable sinus rhythm without any variability due to arrhythmia or respiration. Furthermore, motion of the patient is not an issue in this setup. Another limitation that needs to be discussed is the fact that we did not really measure the applied radiation dose but simply compared the dose indices derived from the CT-scanner’s software. Moreover, regarding the different tube voltages (100 vs. 120 kV) we cannot make conclusion regarding image quality in obese patients due to the fact that the phantom does not account for different subject weight. Further experiments should include these factors, for instance by applying different attenuation phantoms in the setup.

## Conclusion

In conclusion, the presented results do not reveal any relevant differences in vessel diameter, plaque diameter and grade of stenoses between prospectively ECG-triggered high-pitch spiral CT in comparison to retrospectively ECG-gated spiral data acquisition in differently calcified plaques using a motion phantom. While there was reasonable agreement even at higher heart rates (90 bpm) between both examination modes, the presence of motion artifacts at 90 bpm questions the full applicability of the high pitch technique under these circumstances.

## Competing interests

The study was carried out as part of a research agreement with Siemens Healthcare – CT Division -, Forchheim, Germany. TA is an employee of Siemens Healthcare, Forchheim, Germany.

## Authors’ contributions

Study design: TP, ED, PI, TA, CKK, AHM, PB, experiments, measurements: TP, ED, PI, TA, PB, manuscript draft: TP, PI, TA, CKK, AHM, PB. All authors read and approved the final manuscript.

## Pre-publication history

The pre-publication history for this paper can be accessed here:

http://www.biomedcentral.com/1471-2342/14/30/prepub
